# Pathomics Image Analysis of Tumor Infiltrating Lymphocytes (TILs) in Colon Cancer

**DOI:** 10.21203/rs.3.rs-6173056/v1

**Published:** 2025-04-01

**Authors:** Yuwei Zhang, Shahira Abousamra, Mahmudul Hasan, Luke Torre-Healy, Spencer Krichevsky, Sampurna Shrestha, Erich Bremer, Derek A. Oldridge, Andrew J. Rech, Emma E. Furth, Therese J. Bocklage, Justin S. Levens, Isaac Hands, Erich B. Durbin, Dimitris Samaras, Tahsin Kurc, Joel H. Saltz, Rajarsi Gupta

## Abstract

We developed a deep learning Pathomics image analysis workflow to generate spatial Tumor-TIL maps to visualize and quantify the abundance and spatial distribution of tumor infiltrating lymphocytes (TILs) in colon cancer. Colon cancer and lymphocyte detection in hematoxylin and eosin (H&E) stained whole slide images (WSIs) has revealed complex immuno-oncologic interactions that form TIL-rich and TIL-poor tumor habitats, which are unique in each patient sample. We compute Tumor%, total lymphocyte%, and TILs% as the proportion of the colon cancer microenvironment occupied by intratumoral lymphocytes for each WSI. Kaplan-Meier survival analyses and multivariate Cox regression were utilized to evaluate the prognostic significance of TILs% as a Pathomics biomarker. High TILs% was associated with improved overall survival (OS) and progression-free interval (PFI) in localized and metastatic colon cancer and other clinicopathologic variables, supporting the routine use of Pathomics Tumor-TIL mapping in biomedical research, clinical trials, laboratory medicine, and precision oncology.

## 1. Introduction

Colon cancer is a common and deadly form of cancer that is a serious global public health problem whose global incidence is projected to be 3.2 million new cases per year and 1.6 million annual deaths around the world by 2040[1,2]. Screening and surgical intervention are useful if colon cancer is detected at an early stage, but mortality rapidly declines in advanced stages. Fortunately, immunotherapy appears to be a useful treatment option in primary and metastatic colon cancer with microsatellite instability (MSI), creating an urgent clinical need to identify biomarkers to guide treatment selection and improve treatment response. The relationship between host immunity and cancer has been an active area of biomedical research for over a century[3] across the disciplines of oncology, surgery, pathology, immunology, molecular cell biology, pharmacology, and engineering. Numerous studies have revealed that tumor evolution and disease progression are based on close interactions between cancer cells and the host immune system in the tissue microenvironment[4–9], including molecular immune subtypes[10] that range from highly inflamed, immune-active tumors (e.g., IFN-γ dominant) to immune-suppressed, poorly infiltrated tumors (e.g., lymphocyte-depleted and TGF-β dominant). Tumor infiltrating lymphocytes (TILs) are a subset of immune cells that recognize and eliminate cancer cells, leading to significant interest in their characterization as prognostic and predictive biomarkers to improve therapeutic efficacy for oncology[11–13]. TILs present a special opportunity as a biomarker because their assessment is possible in histologic tissue samples stained with hematoxylin and eosin (H&E), which are routinely prepared by clinical and research pathology laboratories.

However, TILs are not routinely assessed in most solid cancers despite the presence of immune cells being useful as predictive biomarkers to stratify patients for therapeutic interventions, including immunotherapy, and safeguard patients from adverse effects[10,14]. Diagnostic microscopic examination of cancer tissue samples is based on characterizing a wide variety of histopathologic features of diseases with a primary focus on Tumor, Node, and Metastasis (TNM) staging. Histologic growth pattern, tumor size, invasion depth, inflammatory infiltrates, necrosis, lymph node involvement, distant metastases, and surgical margins are systematically evaluated, but TILs have yet to be incorporated due to barriers, such as limited time from increased workloads, additional training, and observer variability. Pathomics image analysis tools represent a tremendous opportunity to help pathologists routinely assess TILs in every tissue sample while advancing our understanding of cancer pathobiology to improve treatment and clinical outcomes. A variety of Pathomics methodology has been developed to quantitatively characterize a wide spectrum of histopathologic features of disease to improve diagnostics, prognostication, guide treatment, and discover novel biomarkers[15–18]. One of our major contributions to Pathomics has been the development of a pan-cancer TIL algorithm to map and quantify TILs in digital H&E whole slide images (WSIs) in 13 types of cancer in 2018 and then extended to 23 types of cancer in 2022. At the time, we considered all lymphocytes in a cancer H&E WSI to be a TIL, which was subsequently refined by utilizing cancer segmentation to identify intratumoral TILs in cancer regions consistent with histopathologic terminology. Our efforts to map and quantify TILs at the tissue level has been inspired by the standardized methodology proposed by TIL Working Group of the International Immuno-Oncology Working Group TILs to manually estimate the density of TILs in cancer-associated stroma (sTILs) in H&E glass tissue slides and H&E WSIs for several types of cancer due to their association with improved survival[11,12,19–21].

Since there are currently no standardized approaches for pathologists to quantitatively report the presence of TILs in colon cancer, we developed our Pathomics workflow to provide a simplified view and quantitative assessment of TILs in colon cancer. We utilized our open-source third-generation lymphocyte/TIL deep learning computer vision model in combination with a colon cancer segmentation model developed in-house to generate Tumor-TIL maps for every H&E WSI in our datasets. Our goal with Tumor-TIL mapping is to provide a readily verifiable, immediately interpretable, and quantitative Pathomics data product to routinely evaluate TILs in all H&E WSIs for every colon cancer patient. The combination of Tumor-TIL mapping has revealed a fascinating and complex diversity of intra- and peritumoral TIL-rich and TIL-poor tumor habitats in the colon cancer microenvironment (TME), which are unique in each tissue sample. TILs% is computed to quantify the presence of intratumoral TIL-rich habitats as the fraction of colon cancer tissue occupied by TILs for potential use as a reportable Pathomics biomarker in laboratory medicine to support precision oncology. An area of particular interest for generating Tumor-TIL maps and computing TILs% for every sample is the improvement of treatment success with immunotherapy, where durable treatment responses are still limited to 10–20% of cancer patients[22]. In comparison to the significant manual efforts to distinguish and estimating sTILs and intraepithelial TILs (iTILs) within a local histological niche in a cancer region, our Pathomics workflow is a global ecological survey of tumor immune interactions at the tissue level that is designed for high-throughput and scalable implementation in clinical and research laboratories for screening, cohort discovery, and triage for a comprehensive assessment of sTILs, iTILs, and tertiary lymphoid structures. Related to distinguishing different types of TILs, the development of Immunoscore has been a significant contribution in characterizing TILs in colon cancer, where a quantitative characterization of the density and spatial distribution of CD3 + and CD8 + T cells within the tumor and at the invasive margin[11] has been valuable in predicting clinical outcomes and highlighting the critical role of the tumor immune interactions in the disease progression of colon cancer[23,24].

In our study, our Pathomics workflow mapped the immune landscape of colon cancer and evaluated the prognostic significance of TILs% as a Pathomics biomarker in H&E WSIs from the TCGA colon adenocarcinoma (COAD) dataset and a private dataset from the University of Kentucky (UKentucky), which was specially curated to investigate the relationship between the histological phenomena of tumor budding and obesity[25]. TILs% in colon cancer was evaluated with clinicopathologic correlation and Kaplan-Meier survival analyses to determine its prognostic value to predict overall survival (OS) and progression-free interval (PFI) in localized and metastatic disease based on American Joint Committee on Cancer (AJCC) staging. We also performed novel survival analyses within the context of several clinicopathologic parameters with hypothesis driven, stratified Kaplan-Meier analyses with respect to tumor content in each sample (tumor%), gross tumor size, grading based on histologic cancer differentiation, microsatellite instability (MSS/MSI-L vs MSI-H), and immune subtypes (C1: wound-healing phenotype, C2: IFN-γ dominant profile) in the TCGA COAD dataset. For UKentucky dataset, stratification was based on tumor content, gross tumor size, microsatellite instability, and tumor budding scores. In addition, Tumor-TIL maps for every H&E WSI of TCGA COAD are made publicly available through a web interface (https://rgdpl.bmi.stonybrook.edu/yproj/Tumor-TILs/) for interpretation alongside clinicopathologic and computed Pathomics data (TILs% and tumor%). Our results verify previous observations about the prognostic and predictive value of TILs in colon cancer while emphasizing the value of Pathomics as a reliable, efficient, and scalable solution to contextualize and quantify tumor immune ecology. In light of the rising rates of colon cancer and implications for its potential use in guiding the use of immunotherapy for personalized treatment strategies, we present our Pathomics workflow to demonstrate the feasibility of automating the routine assessment of TILs as a reportable laboratory biomarker for colon cancer patients across a broad spectrum of applications in translational biomedical research, clinical trials, and precision oncology.

### 2. Methods

Two separate deep learning Pathomics models were utilized to map and quantify the presence of colon cancer and TILs in H&E WSIs from the TCGA COAD and UKentucky datasets. The TIL model[18,26], introduced in 2018 and refined as a third-generation model in 2022, was utilized to identify ≥ 2 TILs in 50 $\mu m^2$ tiled image patches, which are stitched together to generate spatial lymphocyte/TILs probability maps for each case. Employing a similar strategy, we developed a colon cancer segmentation model to analyze 87.5 $\mu m^2$ tiled image patches of the same H&E WSIs, which are stitched together to generate a spatial cancer probability map for each case. The spatial probability maps of colon cancer and TILs are merged to generate a combined Tumor-TIL map (Fig. 1a) for every H&E WSI in the TCGA COAD and UKentucky datasets.

### 2.1 Datasets and Pathomics Algorithms

This study evaluates TILs as a biomarker by utilizing the TCGA COAD and UKentucky datasets (Fig. 1b). The TCGA COAD dataset contains data from 458 patients with 411 diagnostic DX1 H&E WSIs, the DX suffix refer to slides used for primary diagnosis. After pathologist review, small biopsy tissue cases and 14 cases with poor image quality/algorithmic prediction were excluded to limit our analyses to 347 DX1 H&E WSIs, predominantly derived from surgical colon cancer resection. We extracted tumor size and histological grade information for these 347 TCGA COAD H&E WSIs from the diagnostic surgical pathology reports made available by the TCGA[27], which are available from the Cancer Digital Slide Archive: https://cancer.digitalslidearchive.org. The UKentucky dataset contains data from 100 patients, where 62 patients were associated with 245 H&E WSIs and multiple slides per case. After 67 non-cancer images were excluded, 178 cancer-containing images were analyzed and consolidated to report an average TILs% per patient for 58 cases in the UKentucky dataset. A private collection of 200 colon cancer H&E WSIs from the University of Pennsylvania (UPenn) was utilized to generate training data to develop the cancer segmentation model, but not included in the analyses due to the limited access to corresponding clinicopathologic and survival data. Every Tumor-TIL map that was generated for all three datasets were manually inspected by a trained pathologist (RG) for quality control (QC) to evaluate colon cancer segmentation and lymphocyte/TIL detection overlaying and/or alongside high-resolution heatmaps on the digital H&E WSIs from these datasets, which are hosted on the open-source Quantitative Imaging in Pathology (QuIP) software platform developed by Stony Brook University and Emory University.

Our Pathomics workflow utilizes our open-source third-generation semi-supervised TIL Inception V4-based model to identify ≥ 2 TILs in 200×200 pixels at 40X magnification to generate spatial lymphocyte/TIL probability maps with a spatial resolution of 50 $\mu m^2$. We developed an in-house supervised ResNet34-based model to detect and segment colon cancer in H&E WSIs. The colon cancer model identifies ≥ 1 cancer cell in 350×350 pixels at 40X magnification to generate a colon cancer probability map. The results from these two separate deep learning computer vision algorithms are merged and threshold at probability > 50% to generate Tumor-TIL maps for each H&E WSI. The colon cancer model (Table 1 and Supplementary Data1) was trained by annotating regions of colon cancer and normal, reactive, dysplastic, and stromal connective tissue in 200 UPenn H&E WSIs. The model was trained, tested, and validated with a 70/20/10 split of 234,679 colon cancer patches and 690,581 non-cancer patches, including examples of 7,205 premalignant dysplasia patches, 128,610 normal epithelium patches, 550,858 stromal connective tissue patches, and 3,908 necrosis patches. Figure 2 is shown as an example of how spatial probability maps are generated for each of the subclasses for the colon cancer segmentation model, except for the lymphocyte/TIL map generated by the third generation TIL model. However, the focus of our attention at this point in our research is based on combining the lymphocyte/TIL map with the spatial probability map of colon cancer only in order to get a global overview of intratumoral and peritumoral by using Tumor-TIL maps.

Table 1. SBU colon cancer detection model

| | Precision | Recall | F1-score |
|---|---|---|---|
| Cancer | 0.95 | 0.99 | 0.97 |
| Dysplasia | 1.00 | 0.96 | 0.98 |
| Epithelium | 0.98 | 0.99 | 0.99 |
| Stroma | 0.98 | 1.00 | 0.99 |
| Necrosis | 1.00 | 0.96 | 0.98 |

**Table 1.** Performance metrics of the SBU colon cancer detection model. The model demonstrates high performance across all categories, including cancer, dysplasia, epithelium, stroma, and necrosis, where precision, recall, and F1-cores exceed 0.95 based on a 70/20/10 split of training, testing, and validation data. Even though our model can perform subclassification of the complex histologic landscape of histologic colon cancer tissue samples as shown in Figure 2, we focus on globally distinguishing cancer regions from non-cancer tissues in order to study the overlap of cancer regions with the spatial lymphocyte probability map to evaluate intratumoral TILs in colon cancer. Every Tumor-TIL map was manually examined by a trained pathologist (RG) during QA. Cancer, normal epithelium, and stroma classification performance is similar to other published colon cancer segmentation models[15], whereas precision and recall values for dysplasia and necrosis are results of model overfitting.

## 2.2 Tumor-TIL maps for TCGA COAD and UKentucky cases

Tumor-TIL maps were generated for all H&E WSIs in the TCGA COAD and UKentucky datasets. Each pixel in the spatial probability heatmaps represents a tiled patch from the corresponding H&E WSI. As shown in Fig. 1, Tumor-TIL maps are generated by amalgamating the outputs from the colon cancer and TIL models, where a probability threshold of ≥ 50% was considered positive, while values < 50% were deemed negative to form the final integrated map. Due to the different patch sizes that are used for tumor and TIL detection, we scaled and aligned the outputs before merging to construct the Tumor-TIL maps. The Tumor-TIL maps employ a consistent color scheme with yellow to denote cancer regions, red to denote TIL-positive patches, and gray to denote non-cancer/non-TIL background tissue (Fig. 1a, Fig. 2 third column, third row). The Tumor-TIL maps are used to evaluate the global abundance and spatial distribution of intratumoral and peritumoral TILs, including tertiary aggregates, for each H&E WSI, which are quite complex and heterogeneous as shown in Fig. 3.

## 2.3 Calculation of tumor and intratumoral TILs

Several quantitative metrics were calculated from Tumor-TIL mapping for each case. Tumor content (tumor%) was calculated per H&E WSI as the number of cancer-positive patches divided by the total number of tissue patches, expressed as:

$$\text{Tumor }\% = \left(\frac{N_{\text{cancer}}}{N_{\text{total}}}\right) \times 100$$

where $N_{cancer}$ represents the number of cancer-positive patches, and $N_{total}$ denotes the total number of tissue patches.

Tissue lymphocytes (%) per H&E WSI was determined by dividing the number of lymphocyte-positive patches by the total number of tissue patches:

$$\text{Tissue Lymphocytes }\% = \left(\frac{N_{\text{lymph}}}{N_{\text{total}}}\right) \times 100$$

where $N_{lymph}$ indicate the number of lymphocyte-positive patches.

Intratumoral TILs% represents the fraction of the cancer tissue infiltrated by TILs, which was calculated as the ratio of positive patches for both cancer and TILs to the total number of cancer patches:

$$\text{Intratumoral TILs }\% = \left(\frac{N_{\text{TILs}}}{N_{\text{cancer}}}\right) \times 100$$

where $N_{TILs}$ is the number of patches positive for lymphocytes in the cancer region.

From the image analysis perspective, the intratumoral TILs metric gauges the proportion of the Tumor-TIL map that appears reddish from the overlap of yellow and red in TIL-positive/cancer-positive pixels. After computing these metrics for all cases, the mean value was used to categorize images as 'high' and 'low in terms of tumor content, as well as 'high' and 'low' for intratumoral TILs%. These groupings were correlated with patient outcomes (Table 2, Supplementary Data2, Supplementary Data3) to evaluate intratumoral TILs as a biomarker with survival analyses stratified by AJCC colon cancer staging, tumor%, gross tumor size, grade by cancer differentiation, microsatellite instability (MSI), and tumor budding in UKentucky.

**Table 2. Tissue distribution**

| Tissue | TCGA COAD (N=347)<br>m±std (%) | UKentucky (N=58)<br>m±std (%) |
|---|---|---|
| Tumor Content (tumor%) | 66.2 ± 21.1% | 31.5 ± 14.2% |
| Tissue Lymphocytes (%) | 13.3 ± 7.6% | 13.3 ± 6.2% |
| Intratumoral TILs (TILs%) | 14.1 ± 8.6% | 28.7 ± 14.1% |

**Table 2.** Tumor-TIL mapping derived metrics for TCGA COAD and UKentucky datasets. The mean values were calculated by summing metrics across all WSIs and dividing by the total number of WSIs.

### 2.4 Survival analyses

We conducted Kaplan-Meier analyses to assess the prognostic significance of TILs% as a tissue-level biomarker in colon cancer[24,28]. We studied the relationship between high and low TILs% with overall survival (OS) by using the total duration from the time of diagnosis or initiation of treatment until death. We also studied the relationship between high and low TILs% with progression-free interval (PFI) by using the duration that a patient lives with the disease without it worsening, both during and after treatment. PFI serves as a preferred endpoint for survival analyses in the context of colon adenocarcinoma clinical trials since it measures the time from primary treatment to the point of disease recurrence[29]. The statistical significance of our findings was evaluated with the log-rank test, which notably does not necessitate prior knowledge of the shape of the survival curve or the distribution of survival times, making it a robust tool for comparing groups in this scenario. Multivariate Cox Regression models[30] were employed to discern whether the presence of these specific features contributed to an increased or decreased risk of survival.

### 3. Results

## 3.1 Defining high and low and intratumoral TILs cohorts in TCGA COAD and UKentucky

The TCGA COAD and UKentucky datasets were appraised to identify possible confounding factors before proceeding with the main analyses to evaluate intratumoral TILs as a prognostic biomarker. The patient population within TCGA COAD exhibited a demographic split of 48.1% females and 51.9% males, whereas UKentucky is comprised of 55.2% females and 44.8% males. TILs% is similar in the males and females from both datasets. The TCGA COAD and UKentucky datasets featured a greater number of patients with localized disease (AJCC stage I and stage II) as opposed to metastatic disease (AJCC stage III and stage IV). In terms of microsatellite instability (MSI) status, a substantial proportion of the TCGA COAD cases were labeled as microsatellite stable (MSS) or low microsatellite instability (MSI-L), overshadowing the number of cases with high microsatellite instability (MSI-H). Similarly, UKentucky cases were overwhelmingly labeled as MSS or MSI-L with only one MSI-H case. In addition, UKentucky included tumor budding scores that was not assessed in TCGA COAD (Table 3).

**Table 3. Patient characteristics**

| Characteristics | TCGA COAD<br>N = 347 | UKentucky<br>N=58 |
|---|---|---|
| Age, median (interquartile range) | 66 (59-78) | 63 (55-75) |
| Sex | | |
| Male | 180 (51.9%) | 26 (44.8%) |
| Female | 167 (48.1%) | 32 (55.2%) |
| Stage | | |
| I | 57 (16.4%) | 8 (13.8%) |
| II | 141 (40.6%) | 24 (41.4%) |
| III | 95 (27.4%) | 20 (34.5%) |
| IV | 45 (13.0%) | 6 (10.3%) |
| NA | 9 (2.6%) | NA |
| Microsatellite instability (MSI) | | |
| MSS | 214 (61.7%) | 51 (88.0%) |
| MSI-L | 68 (19.6%) | 6 (10.3%) |
| MSI-H | 60 (17.3%) | 1 (1.7%) |
| NA | 5 (1.4%) | NA |
| Gross Tumor Size | | |
| Median (min, max) | 4.8 (0.1-15) | 5 (1.2-21) |
| Tumor Budding Score | | |
| G1 | NA | 23 (47.9%) |
| G2 | NA | 14 (29.2%) |
| G3 | NA | 11 (22.9%) |
| Histological Grade | | |
| Well-differentiated | 20 | NA |
| Moderate-differentiated | 237 | NA |
| Poor-differentiated | 74 | NA |
| NA | 16 | NA |

| | | |
|---|---|---|
| Overall survival (OS.month) | | |
| Median (min, max) | 21.3 (0-150.07) | 45.2 (8.5-79.8) |
| Progression free interval (PFI.month) | | |
| Median (min, max) | 18.3 (0-150.07) | NA |

**Table 3.** Demographics and clinicopathologic features of TCGA COAD and UKentucky datasets. OS.month is the interval commencing from diagnosis and extending to death or loss to follow up. PFI.month reflects the clinical interval of time with no tumor-associated event (e.g., progression of disease, local recurrence, distant metastasis, new primary tumor, or death without new tumor event). Gross tumor size in the tissue removed from after surgery. Additional UKentucky data include tumor budding scores that refer to the presence of individual cells or small clusters of tumor cells at the invasive front of the tumor.

As described in 2.3, the cancer regions within all H&E WSIs from TCGA COAD and UKentucky were identified by using the SBU colon cancer segmentation model to calculate tumor content (tumor%) per WSI. The mean tumor% in TCGA COAD was 66.2%, whereas UKentucky was 31.5%. Interestingly, the mean value of the total tissue lymphocytes was 13.3% in both datasets. However, intratumoral TILs% is quite different between TCGA COAD and UKentucky due to the influence of the cancer region area. In TCGA COAD, the tumor% in WSIs is almost double in comparison to UKentucky WSIs.

Since TILs represent how much of the cancer regions are infiltrated as TILs, the mean TILs% in TCGA COAD is 14.1%, which is approximately half that of UKentucky with 28.7%. Further QA showed that the tumor% is the only variable affecting intratumoral TILs% since the overall total tissue area and total lymphocyte% are very similar in both datasets. However, distribution of tumor% within each dataset appears similar in relation to AJCC stages. There is also a significant association between TILs% and AJCC stages I and II. Even though we report population level comparisons between high and low TIL groups in TCGA COAD, our results need to be interpreted within the context of the Tumor-TIL maps themselves, which display much more nuanced information in terms of spatially heterogeneous and complex tumor immune interactions that are immediately evident in each case (Fig. 3).

In the TCGA COAD dataset, we categorized patients into distinct groups based on high or low TILs%. After this initial stratification, we further stratified cases based on AJCC staging to create separate cohorts for stages I-II localized disease and stages III-IV metastatic disease. This approach allows us to examine the interplay between high and low TILs% in early and late-stage cancer. Notably, stages III-IV represent metastatic disease, which is often associated with a significant reduction in survival. Applying the same methodology, we classified subgroups based on microsatellite instability (MSI) status, encompassing MSI-H and MSS/MSI-L categories. These distinctions arise from mutations in DNA mismatch repair genes and are identified in 10–15% of sporadic colorectal cancers[31]. Gross tumor size was utilized to split the TCGA COAD dataset to evaluate the effect of high and low TILs%. Additionally, we stratified and defined cohort based on high and low tumor% in WSIs to delve deeper into the exploration of the role of the complex dynamics of immune interactions within cancer while exploring potential confounding effects of tumor bulk on TILs%. To do this, we defined high and low tumor% to evaluate the effect of high and low TILs% in each group.

We also used the same methodology to categorize the UKentucky dataset into high and low TILs%, further stratified by AJCC stages or tumor% in WSIs. Kaplan-Meier analyses are performed with OS only. Furthermore, we used the additional clinical feature in the UKentucky dataset to stratify into grade 1 (G1), grade 2 (G2), and grade 3 (G3) by tumor budding scores. Based on Ueno method[32], tumor buds smaller than 5 tumor cells in one field (one hotspot) at the invasive front were counted under 20X objective lens, where G1 represents 0–4 tumor buds, G2 is defined as 5–9 tumor buds, and G3 is identified as more than 10 tumor buds.

### 3.2 Relationship between TILs%, AJCC stages, MSI status, gross tumor size, molecular immune subtypes, and survival

TILs% is significantly associated with extended OS and enhanced PFI within the TCGA COAD cohort (Fig. 4a, Supplementary Data4). To further delve into the prognostic utility of TILs as a biomarker in colon cancer, our analyses included the stratification of the cohort into distinct categories based AJCC stages. Stages I-II represent localized disease group, where stage I represents invasion into the submucosa/muscularis propria (T1/T2) and stage II signifies cancer growth into the outermost layers of the colon (T3) or through the wall of the colon (T4) without nodal (N0) or distant metastases (M0). Stages III-IV represent metastatic disease, where stage III represents T1-T4 with nodal metastases (N1-N2) and M0 and stage IV signifies T1-T4, N0-N2, and distant metastases (M1). After stratifying the cohort into stages I-II and stages III-IV, each cohort was split into high and low TILs% for Kaplan-Meier analyses. The survival analyses show that patients with high TILs% in stages I-II and stages III-IV are associated with the better survival benefit after approximately 4 years. Interestingly, patients with high TILs% also exhibit more favorable disease control in stages I-II and stages III-IV (Fig. 4b-c).

We also conducted an in-depth examination of the relationship between TILs% and MSI, which is critical parameter that is routinely assessed in colon cancer patients per clinical guidelines[14,33] (Fig. 4d-e). MSI status evaluates defective DNA mismatch repair (MMR) genes (e.g. MLH1, MSH2, MSH6, or PMS2), where MSI-L indicates instability at one site and MSI-H indicates instability at two or more sites. MSS represents no abnormalities. After stratifying the groups in MSI-H and MSS/MSI-L, each cohort was split into high and low TILs% for Kaplan-Meier analyses to show that high TILs% is associated with better OS and PFI in MSS/MSI-L. For MSI-H patients, high TILs% appears to be associated with PFI and no association with OS in a very limited dataset, which suggests a possible role for TILs% as a biomarker for disease control.

In addition, Kaplan-Meier analyses were utilized to evaluate the prognostic significance of TILs% with respect to histological grade in relation to OS and PFI. Cancer grading represents the level of differentiation of cancer cells to describe how closely their appearance resembles epithelial cells in normal colonic tissue. Well-differentiated to moderately-differentiated indicates that the growth pattern of cancer cells and their cytology resemble the appearance of normal colonic epithelial tissue cells in terms of tissue architecture, glandular structural configuration, functional orientation and polarity, and cytomorphology. In contrast, poorly-differentiated colon cancer lacks a resemblance to normal colonic tissue architecture and cells, typically appearing as sheets of cells without gland formation. Our analyses also revealed a positive correlation in both subgroups between OS and PFI, indicating that better outcomes were observed in patients with high TILs% (Fig. 5a-b).

These findings highlight the crucial role of immune infiltration in shaping the tumor microenvironment and influencing clinical outcomes. Due to availability of molecular immune subtype based on bulk gene expression data for TCGA COAD specimens, we also correlated TILs% in an exploratory attempt to further investigate the prognostic significance of high TILs% with respect to molecular subtype. Seeing the diversity of the magnitude and spatial distribution of tumor immune responses within the complex histologic landscape of colon cancer[34] suggests that there is underlying difference in TIL populations composed of functionally specific types of immune cells (CD4 + and CD8 + T-cells, B-cells, and NK cells), which are likely based on molecular differences due to gene expression. The molecular subtypes in the TCGA COAD dataset include C1 (wound-healing phenotype), C2 (IFN-γ dominant profile), C3 (inflammatory subtype), C4 (lymphocyte depletion), C5 (immunologically quiet), and C6 (TGF-β signaling). Since most of the TCGA COAD cases are either classified as the C1 or C2 molecular immune subtype (Supplementary 5), so we limited the Kaplan-Meier analyses to evaluate TILs% to these two groups (Fig. 5c-d).

## 3.3 Relationship between tumor% and TILs% with Kaplan-Meier analyses

AJCC colon cancer staging is determined by the extent of cancer invasion through the layers of the colon wall, not the size of the tumor[35,36] that plays a more major role in TNM and AJCC staging for most other solid tumors to predict prognosis, recurrence, and survival. However, colon cancer biology is complex, and we leveraged Pathomics image analysis to pursue a novel opportunity to explore the effect of tumor cellularity/size via tumor% as a surrogate parameter in H&E WSIs to investigate the effect of TILs% as a biomarker in colon cancer with respect to OS and PFI. Tumor% was used to split the cohort into high and low tumor% for Kalan-Meier analysis, where low tumor% is associated with better survival outcomes and disease control (Fig. 6a). In the low tumor% group, high TILs% was associated with enhanced OS and PFI (Fig. 6b). In high tumor%, high TILs% is initially associated with increased OS for approximately 4 years while being associated with enhanced PFI (Fig. 6c). Despite the limitations of these analyses due to the assumption that increased tumor% in WSIs is generally unfavorable, high TILs% still appears to be valuable as prognostic and potentially predictive biomarker, which may be useful for guiding treatment with long-term efficacy.

TILs% appears to be a mostly independent biomarker with respect to tumor burden, where tumor heterogeneity and cancer pathobiology are more likely to be different in larger tumors in comparison to smaller, earlier stage tumors. While invasion through colonic layers determines stage, these observations emphasize the importance of examining the context of TILs within the broader tumor microenvironment to understand their role in tumor progression and prognostication. However, this is exploratory at best due to inherent limitations in the availability H&E WSIs of tissue samples that were constrained to a single diagnostic slide per case. The amount of tumor heterogeneity within the context of tumor size and its effects on the abundance and distribution of TILs remains mostly unknown in this study. However, our Pathomics workflow is capable of surveying the variability of tumor immune interactions across multiple diagnostic slides per case to evaluate TILs within the context of tumor content, which will be more thoroughly characterized in subsequent studies. Despite these constraints, evaluating TILs% with respect to tumor burden enhances the robustness of our Pathomics workflow with additional Pathomics parameters to deepen our understanding of tumor-immune dynamics.

To further investigate the effect of tumor burden on the complexity of tumor-immune interactions, we also analyzed the relationship between TILs% and gross tumor size that was extracted from diagnostic pathology reports. Even though gross tumor size is not utilized in staging either, the state of tumor progression and potential for tumor heterogeneity might be ascertained for prognostication to some extent (Fig. 6d-e). We stratified cases into large and small categories based on mean gross tumor size to evaluate high and low TILs% in each cohort. Kaplan-Meier analyses demonstrated that patients with high TILs% in both large and small tumors exhibited improved OS and PFI, highlighting the potential value of TILs% as a Pathomics biomarker.

### 3.4 Validation in the UKentucky dataset with additional Kaplan-Meier analyses stratified by tumor budding scores

High and low TILs% was defined by the mean value in the UKentucky dataset to perform a series of Kaplan-Meier analyses limited to OS for comparison to the TCGA COAD dataset. High TILs% displayed a positive relationship with OS (Fig. 7a, Supplementary Data6), followed by stratifying the UKentucky dataset into the localized stages I-II and metastatic stages III-IV disease subgroups. Despite the small number of patients in each subgroup, high TILs% was associated with enhanced OS in stages I-II (Fig. 7b). Low TILs% in stages III-IV was not observed with improved OS (Fig. 7c). The mean value of the gross linear tumor size was used to split the cohort into small and large tumors and explore the effect of high and low TILs%. TILs% is associated with improved OS in both small and large tumors, with the benefit becoming more pronounced in larger tumor size, whereas it is initially less evident in small tumors. (Fig. 7d-e). After stratifying into high and low tumor%, high TILs% in the low tumor% subgroup also appears to be associated with improved OS with a similar trend in the high tumor% subgroup (Fig. 7f-g). Evaluating TILs% in the context of MSI status was also limited since most patients were MSS or MSI-L with one MSI-H case, where our analysis showed that TILs% are associated with enhanced OS in the MSS/MSI-L group (Fig. 8a).

Even though the UKentucky dataset is limited in terms of the number of cases, there were special opportunities to explore the role of TILs% as a biomarker within the setting of tumor budding. Tumor budding is an important prognostic factor for colon cancer, but its association with TILs is unknown. Tumor buds are characterized by the presence of 1–4 malignant cells at the invasive edge of the tumor, where G1 was used to represent low-grade with < 5 buds, G2 for intermediate-grade with 5–9 buds, and G3 for high-grade with ≥ 10 buds. Since tumor budding in colon cancer is consistently associated with lymph node and distant organ metastasis, local recurrence, and poor OS independent of TNM staging[36], we used this examined the relationship of TILs% with tumor budding. We evaluated the effect of high and low TILs% in the context of G1, G2, and G3 scoring, where our analyses showed that high TILs% is associated with improved OS in G1 with a gradually diminishing survival benefit for high TILs% in the G2 and G3 subgroups (Fig. 8b-d).

### 3.5 Multivariate Cox regression analyses of TILs% as a Pathomics biomarker in colon cancer

As shown in (Fig. 9), Multivariate Cox regression analyses were performed in the TCGA COAD dataset to discern whether the presence of specific clinicopathologic features are associated with an increased or decreased risk of survival. Forest plots of the hazard ratios for OS and PFI show the potential survival benefit of TILs, where high TILs% is associated with improved PFI ($p = 0.016$) suggesting better disease control in cases with TILs. In comparison, metastatic disease in AJCC stages III-IV show significantly higher hazard ratios compared to stages I-II for both OS and PFI ($p < 0.001$). Tumor% also emerges as a significant factor for OS ($p = 0.004$) and for PFI ($p = 0.05$) due to its general relationship with increased tumor cellularity, growth rate, and relative size on a given image, whereas MSI status and tumor size are not significant predictors for either outcome.

Multivariate Cox regression analysis was conducted on the UKentucky dataset to evaluate the association of clinicopathologic features with OS. The forest plot (Fig. 10) indicates a potential survival benefit of high TIL% ($p = 0.282$), though not statistically significant. AJCC stages III-IV exhibit an increased hazard ratio compared to stages I-II ($p = 0.46$), suggesting a trend toward poorer survival outcomes. Tumor% and tumor size do not show significant associations with OS ($p = 0.511$ and $p = 0.612$, respectively). Similarly, tumor budding score does not emerge as a strong predictor of survival ($p > 0.5$ for all comparisons).

### Discussion

The Pathomics workflow in this study was designed to perform a histologic survey of tumor immune ecology to gain global insight into the immune landscape of colon cancer. Tumor-TIL maps provided a fascinating view into the complexity and diversity of intra- and peritumoral TIL-rich and TIL-poor habitats in the TME of colon cancer, which are unique in every tissue sample. These observations led to computing TILs% as a parameter to quantitatively describe the presence and extent of intratumoral TIL-rich habitats and evaluating its prognostic significance and robustness as a Pathomics biomarker for precision oncology. We presented the (1) development of a novel Pathomics workflow that utilizes two deep learning algorithms to identify colon cancer and lymphocytes/TILs in H&E WSIs to (2) routinely generate Tumor-TIL maps as novel Pathomics data products for every H&E WSI in two datasets and (3)

evaluate the prognostic value of TILs% as a prognostic Pathomics biomarker in colon cancer with clinicopathologic correlation and hypothesis driven Kaplan-Meier analyses.

Our study shows that high intratumoral TILs% appears useful for predicting improved overall and progression-free survival in local and metastatic disease and within the context of clinicopathologic variables, such as histologic grade, MSI status, tumor burden per sample, gross tumor size, and tumor budding. Survival analyses were systematically performed with OS and PFI, when possible, and stratified survival analyses were based on hypotheses inspired by real world clinical scenarios to investigate the potential value of TILs% as a prognostic Pathomics biomarker for precision oncology. In related efforts in breast cancer, TILs% was computed and evaluated as a biomarker with multivariate Cox regression and survival analyses stratified by staging, expression of hormone receptors, and molecular subtype[37,38]. In this study, we wanted to explore the significance of TILs% in additional scenarios, such as the effect of TIL% in cases with high versus low tumor content in the tissue sample and compare that to TIL% in cases with high versus low gross tumor size. These types of analyses are typically not performed in colon cancer since size is not a major determinant of TNM staging, which is dependent on depth of invasion through the layers of the colonic wall. Similarly, tumor budding has become a biomarker with gaining popularity in colon cancer, but their assessment is not routinely performed or typically available for analysis to evaluate biomarkers, motivating our investigation about the association of TILs% with tumor budding scores to demonstrate proof of principle. These types of hypothesis driven scientific inquiry also motivated the correlation of TILs% with molecular immune subtype from gene expression as an additional way to evaluate the prognostic significance of TILs in colon cancer.

Extensive work by the TIL Working Group of the International Immuno-Oncology Working Group has shown that higher densities of sTILs in cancer-associated stroma are associated with better survival outcomes in several types of cancer[11,12,19–21]. In related efforts to characterize tumor immune ecology, immunohistochemistry (IHC) was utilized to evaluate CD3 + and CD8 + T-cell lineage TILs in the central tumor and invasive leading edge as spatially distinct regions of the TME, which led to the development of Immunoscore[23,24]. The importance of these approaches in bringing attention to TILs as a routine clinical biomarker cannot be understated, where Immunoscore with IHC in colon cancer was recently validated for predicting survival risk[39]. However, distinguishing sTILs and intraepithelial TILs (iTILs) with H&E and/or IHC within the complex histologic landscape of the tumor microenvironment (TME) is a challenging endeavor subject to observer, incorrect categorization, and sampling bias[40]. The powerful capabilities of automated Pathomics methodology can help address this unmet clinical need by modernizing diagnostics and prognostication and supporting biomarker discovery for precision oncology.

Numerous Pathomics tools have been developed to perform detection, segmentation, and classification of tissues, glands, and multiple cell types in several types of cancer to perform histologic subclassification based on architectural growth pattern and quantification of various histologic features, such as immune infiltration, fibrosis, necrosis, tumor budding, and biomarker expression with

IHC[15,16,18,26,41–48]. Our work builds on previous efforts to assess TILs with sophisticated deep learning computer vision algorithms[15–18] amongst a growing ecosystem of innovative Pathomics tools with the capability to predict clinical outcomes, mutations, abnormal molecular pathways, and treatment response directly from H&E WSIs, which are all being actively incorporated into foundation models and combined with large language models (LLMs)[49–52]. Our Pathomics workflow is designed to be conceptually simple for visualizing tissue level immuno-oncologic interactions and compute TILs% in the colon cancer TME, since our goal is to routinely perform Tumor-TIL mapping and TILs% quantification for use as a reportable laboratory biomarker for research and clinical applications in precision oncology. A particular strength of our Pathomics workflow is that every Tumor-TIL map is readily verifiable by histology alongside and/or overlaid on the corresponding H&E WSI.

Tumor-TIL maps show the global abundance of all TILs, consisting of sTILs, iTILs, and tertiary lymphoid structures (TLS) within the context of their intra- and peritumoral spatial distribution in the colon cancer TME, which consists of both malignant epithelium and cancer-associated stroma. In addition to being rapidly interpretable for evaluating the overall abundance of TILs within the histologic context of spatially distinct intra- and peritumoral TIL-rich and TIL-poor tumor habitats, Tumor-TIL mapping can guide the selection of spatially distinct ROIs for sTIL/iTIL quantification and/or select cases for scoring functional subtypes of TILs with IHC markers. Computing TILs% as the amount of cancer occupied by intratumoral TILs is also analytically straightforward for simplifying the preliminary assessment of TILs before further subclassification as sTILs, iTILs, and TLS. This is similar in a manner to how most IHC biomarkers are reported as a percentage of their expression by the overall amount of cancer on a glass tissue slide/WSI and then further evaluated by the magnitude and pattern of their expression within specific cells of interest. Therefore, we also envision Tumor-TIL maps being utilized to guide sampling for downstream genomic and imaging studies (e.g., sequencing, gene expression, multiplexed immunofluorescence, MALDI-TOF) by the density of TIL infiltrates in TIL-rich and TIL-poor areas in the intratumoral and peritumoral compartments of the colon cancer TME.

The TCGA COAD dataset contains mostly balanced demographics and clinical profiles, where the majority of all patients (57%) presented with localized disease (stages I-II) and 48.1% of patients were female with a mean age of 66 years. Additionally, 81.3% of the cases were classified as MSS or MSI-L, consistent with the predominance of these molecular subtypes in colon cancer. Despite its limited sample size, the UKentucky dataset serves as a valuable real-world external validation cohort and an important opportunity to investigate the role of TILs within the context of tumor budding since it is from a study that was originally curated to investigate the association of obesity with histological tumor budding. The UKentucky also exhibits similar distribution for gender, AJCC stages, MSI status, and gross linear tumor size in comparison to the TCGA COAD dataset, allowing us to utilize our Pathomics workflow to perform multivariate Cox regression and stratified Kaplan-Meier analyses to confidently evaluate the association of TILs% with overall and progression-free survival in comparison to TCGA COAD.

It was also reassuring that the average of total tissue lymphocytes% was the same in TCGA COAD and UKentucky datasets at 13.3% (Table 2). This consistency suggests that there might be a baseline level of lymphocytic infiltration in colon cancer tissue samples, where notable differences emerge after computing the tumor% and TILs% to characterize the extent of the colon cancer microenvironment that is infiltrated by TILs. Specifically, the tumor content in the UKentucky dataset is approximately half of that in the TCGA COAD cohort, leading to TILs% being nearly doubled even though the average total lymphocytes% is 13.3%. The distribution of cases remained consistent across subgroups, as demonstrated in (Supplementary Data 1 and Supplementary Data 2), where TILs% distribution was comparable across different subgroups defined by clinicopathologic parameters. Even though we presented aggregated survival analyses by comparing AJCC stages I-II for localized disease to metastatic disease in stages III-IV due to statistical considerations *a priori*, we also included analyses where TILs% were comprehensively evaluated as a biomarker in each AJCC stage from I to IV (Supplementary Data 3 and Supplementary Data 4) to provide insight into statistically underpowered trends in limited sample sizes to show the value of TILs% as a biomarker per AJCC stage I to IV in comparison to results from previous and parallel studies.

Our series of stratified Kaplan-Meier analyses in TCGA COAD utilizing both overall and progression-free interval analysis show that high TILs% appears to have independent prognostic utility, regardless of clinical stage, differentiation, gross linear tumor size, and MSI status. When TILs% are incorporated in the context of tumor content and tumor budding scores, TILs still retained their value as a prognostic biomarker. Even though the datasets are relatively limited after stratification, the findings remain interpretable and relevant to demonstrate the utility of TILs% in predicting survival and disease progression. In terms of exploring how the prognostic utility of TILs% can be confounded by tumor size, we highlighted the novelty of analyzing TILs in the context of both gross linear tumor size and microscopic size/area based on tumor content in WSIs since tumor size may affect the extent of tumor (immune) heterogeneity that may have value in predicting disease recurrence and progression. The results of our study are consistent with the findings of multiple studies that have shown prognostic significance of TILs with methods like Immunoscore[11,19,20,53]. The international validation of the consensus Immunoscore provided a standardized framework[11] for evaluating TILs in colon cancer by calculating the mean densities of CD3 + and CD8 + T-cells in the central tumor region and tumor invasive margin, demonstrating its robust prognostic value across cohorts. Additionally, pooled analyses of cases from large clinical trials such as SCOT and IDEA-HORG[53] have further solidified the predictive and prognostic utility of the Immunoscore in stage III colorectal cancer. Novel Pathomics methods [23] have been recently published to quantify iTILs and sTILs densities by calculating the number of TILs per square millimeter (1 $mm^2$) within tumor regions or stromal areas adjacent to the tumor[48], which are also consistent with the large body of work demonstrating the prognostic value of TILs.

Our Pathomics workflow is designed to be widely accessible, cost effective, and easily implemented for evaluating TILs in routinely available H&E WSIs to complement routine diagnostic histopathologic evaluation and survey the broad interplay of immuno-oncologic interactions in small and large datasets from diverse patient populations. This work is a proof of concept that demonstrates the value of tissue level characterization of TILs as a starting point for further analyses to examine reproducibility with other readily available colon cancer segmentation and immune cell detection tools. Similarly, our analyses can be compared to analyses with more advanced tools that utilize histologic subclassification of different regions to further separate colon cancer from cancer-associated stroma and cellular characterization of tumor and immune cells with nuclear segmentation and classification tools. Even though we are potentially underrepresenting the additional complexities of the tumor microenvironment, such as the presence of other types of immune cells, stromal reactions, and microvasculature proliferation, localized cellular approaches also introduce bias that will need thorough vetting before clinical implementation. Nonetheless, these approaches and Pathomics tools are all complementary to each other and we envision utilizing a suite of algorithms to comprehensively characterize tumor immune interactions by using data from H&E, IHC, and multiplex immunofluorescence images from multiple datasets in the very near future alongside correlation with laboratory, genomic, transcriptomic, metabolomic, and pharmacologic data from clinical trials.

We believe that personalized precision immuno-oncology will require even greater collaboration in the computational pathology community and multimodal data integration to develop novel Pathomics methodology to harness the transformative potential of Pathomics in advancing cancer diagnostics and treatment. We hope that our Pathomics workflow serves as a provocative example of how robust Pathomics methodologies can be leveraged to offer a reproducible, fast, and efficient method to evaluate tumor immunogenicity, which can support personalized treatment planning by guiding the selection and use of immunotherapy in novel treatment strategies while paving the way for deeper insights into cancer pathobiology. Future work will focus on further validating our findings in independent and larger cohorts to enhance our understanding of the role of TILs in colon cancer and to refine our Pathomics methodology for clinical translation and adoption in precision oncology and personalized cancer treatment.

### Declarations

#### Competing Interests

Rajarsi Gupta, Tahsin Kurc, and Joel H. Saltz are co-founders for Chilean Wool LLC. All other authors declare no financial or non-financial competing interest.

## Author Contribution

Y.Z. and R.G. conceived this study. R.G., Y.Z., S.A., M.H., D.M., T.K., and J.S. contributed to the development of the lymphocytes and tumor segmentation models. R.G., Y.Z., T.K. L.T.H., and S.K. contributed to the selection, acquisition, and processing of the patient samples. R.G., Y.Z., and S.S. contributed to the development and deployment of the website for hosting the Tumor-TIL maps for the public TCGA dataset. R.G., Y.Z., D.O., A.J.R., E.E.F., J.S.L., I.H., E.B.D., and T.J.B. contributed to the patient selection and clinical data acquisition. R.G. and Y.Z. wrote the manuscript with revisions from all authors. All authors approved the final version of the manuscript and agreed to submission. R.G. supervised the work.

## Acknowledgement

This work was supported by the National Institutes of Health (NIH) and National Cancer Institute (NCI) grants UH3-CA22502103, U24-CA21510904, 1U24CA180924-01A1, 3U24CA215109-02, and 1UG3CA225021-01, as well as generous startup support from Stony Brook Foundation (Project 1181919/Award 97222 and Project 1189401/Award 100377) and private support from Betsy Barton and Bob Beals.

## Data Availability

1. Clinicopathological data and images are from The Cancer Genome Altas (TCGA) Colonic Adenocarcinoma (COAD) dataset2. Pathomics Image Analysis of Tumor Infiltrating Lymphocytes (TILs) in Colon Cancer Tumor-TIL maps of TCGA COAD H&E WSIs:
https://rgdpl.bmi.stonybrook.edu/yproj/Tumor-TILs/

## Figures

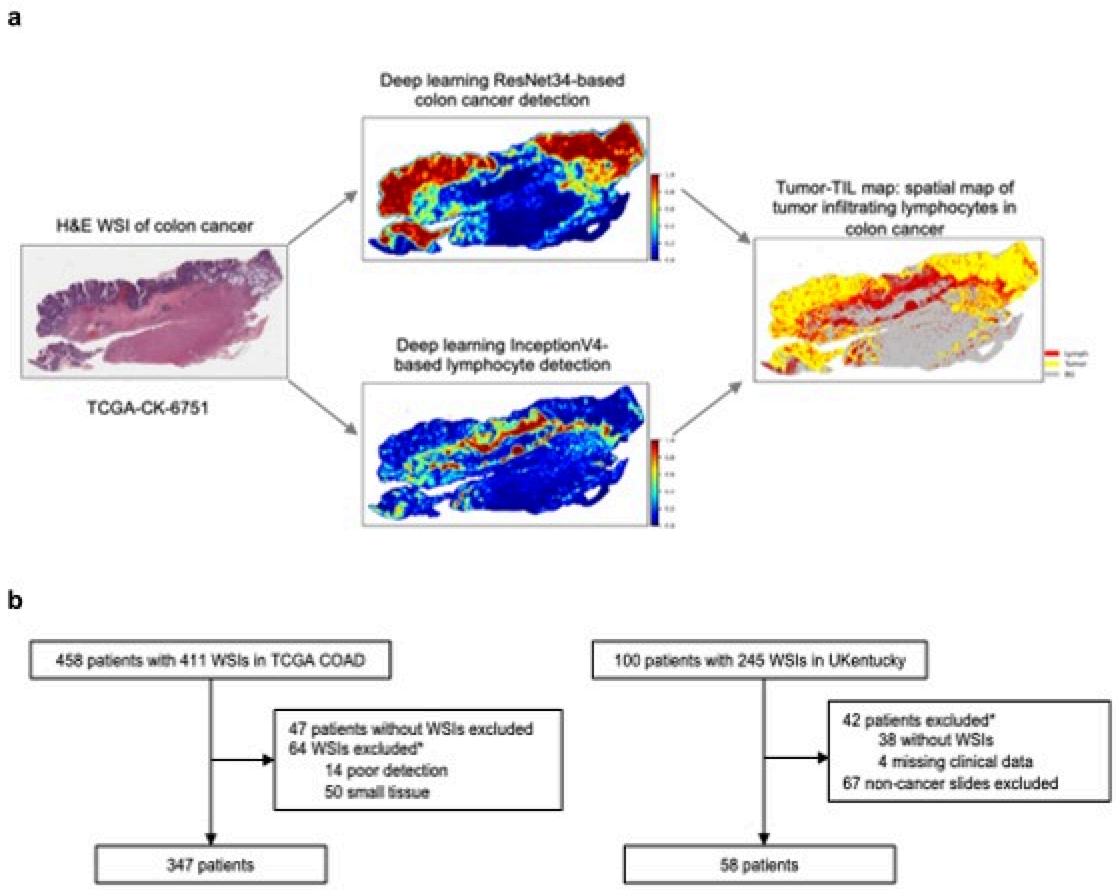

### Figure 1

Colon cancer Tumor-TIL mapping workflow and datasets. a) Deep learning Pathomics models to spatially map TILs in colon cancer H&E WSIs. The top panel shows the spatial probability heatmap of colon cancer at 87.5 $\mu m^2$ resolution with non-tumor tissue in blue. The bottom panel represents the spatial probability heatmap of lymphocytes/TILs at a resolution of 50 $\mu m^2$ with non-lymphocyte tissue in blue. By combining the colon cancer and lymphocyte spatial probability heatmaps and utilizing a probability threshold of ≥ 50% for positive areas, Tumor-TIL maps are generated as tangible Pathomics data products to visualize and quantify the percent of the cancer region that is occupied by intratumoral TILs and evaluate their spatial distribution (colon cancer colored yellow, TILs colored red, and non-cancer/non-lymphocyte background tissue colored gray). b) Datasets of TCGA COAD and UKentucky H&E WSIs were utilized in this study. Example shown is TCGA-CK-6751 from TCGA COAD.

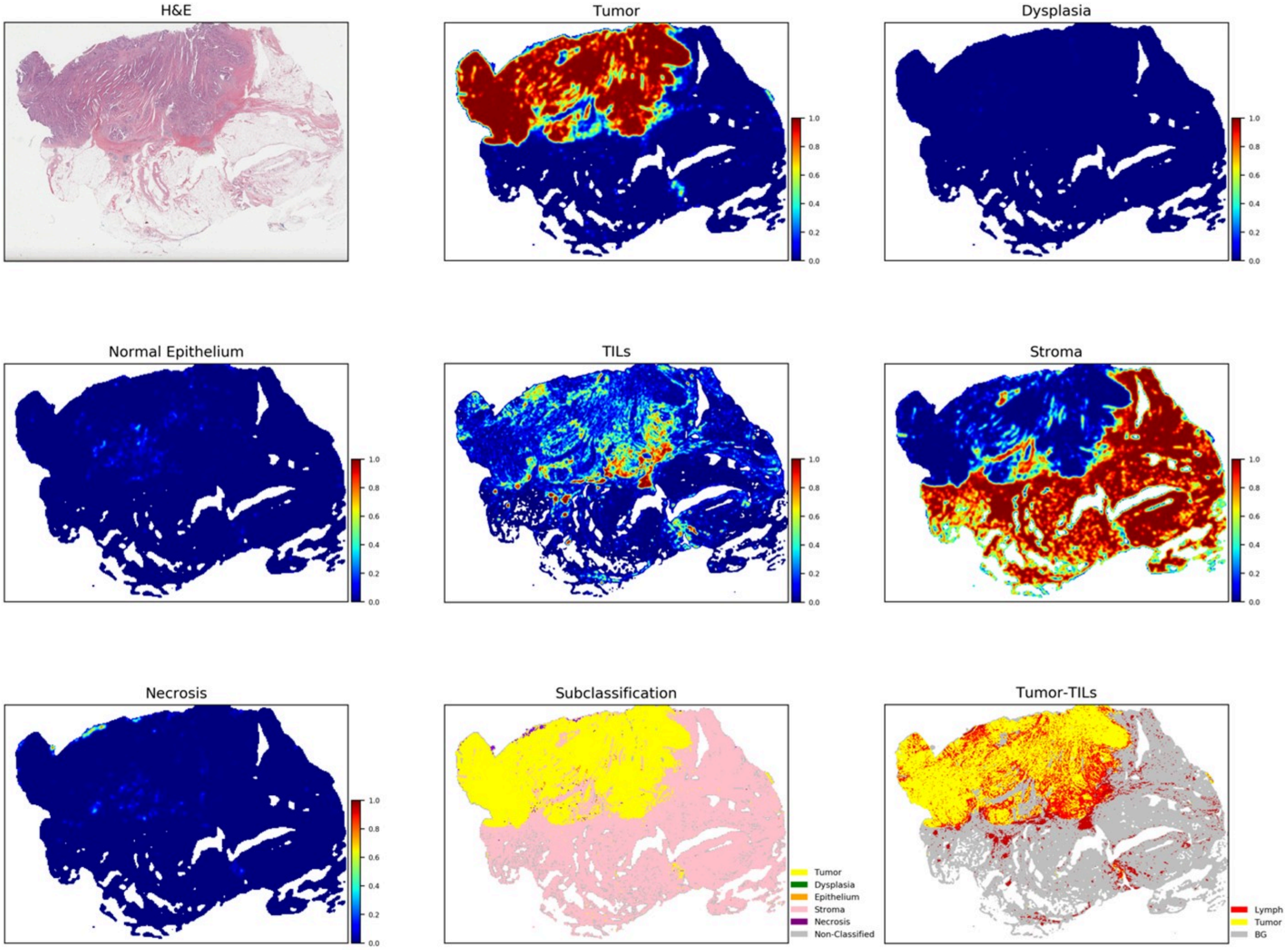

### Figure 2

Example of the colon cancer segmentation and subclassification of histologic sub-compartments. Spatial probability maps are generated for colon cancer, premalignant dysplasia, normal epithelium, stromal connective tissue, and necrosis patches, where the lymphocyte/TIL probability map is generated by the third-generation TIL model. This study focuses on evaluating the value of Tumor-TIL for a global overview about the abundance and spatial distribution of intratumoral and peritumoral TILs. H&E WSI: TCGA-G4-6627-DX1.

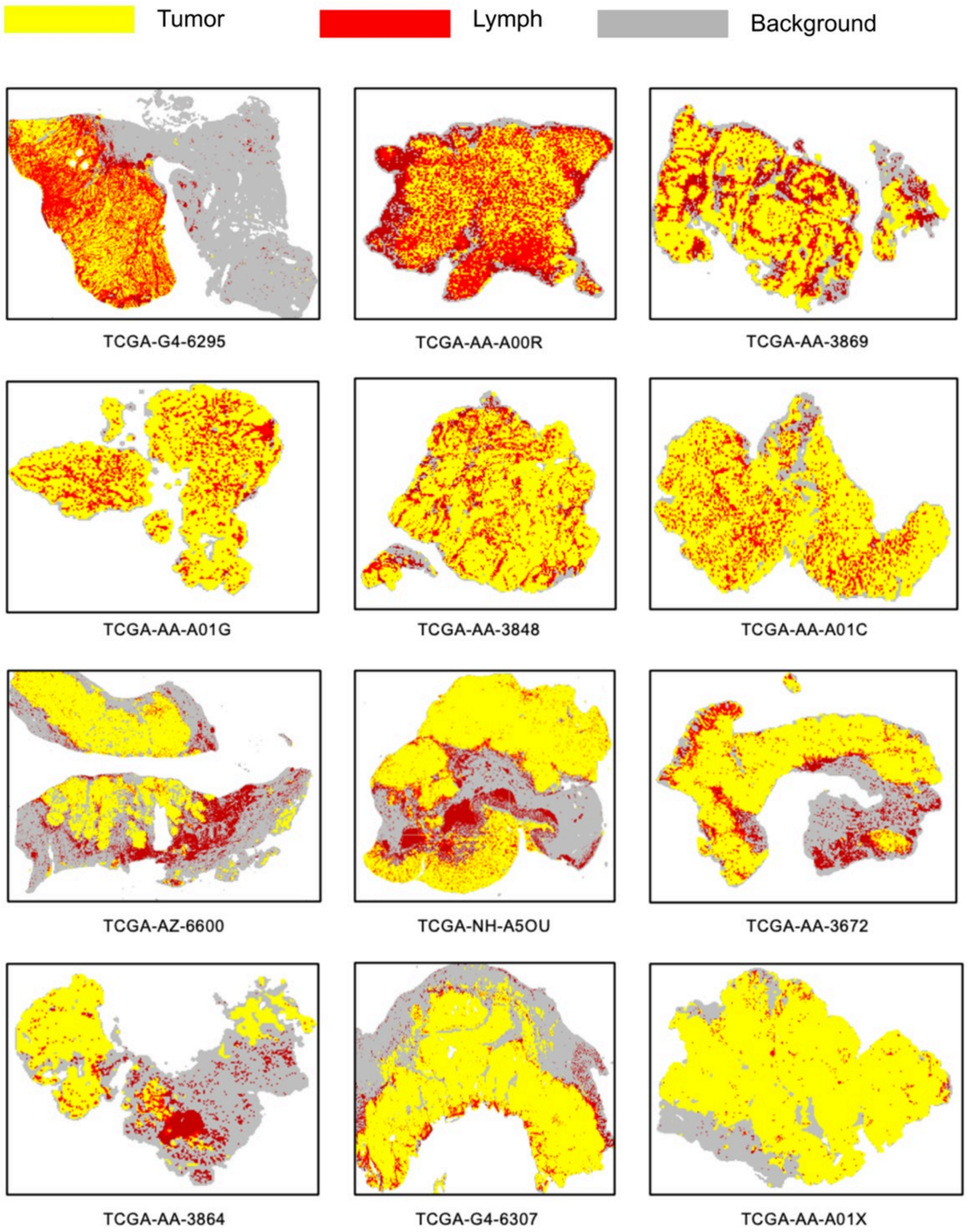

### Figure 3

Heterogeneity of tumor immune interactions. Tumor-TIL maps offer a straightforward and visually intuitive means of illustrating the abundance and spatial distribution of TILs for each DX1 H&E WSIs from the TCGA COAD dataset. The magnitude and spatial distribution of intratumoral and peritumoral TILs is unique in each colon cancer sample. Going from the top right to the bottom left panel, the colon cancer microenvironment can be immunologically hot with large intratumoral TIL infiltrates to immunologically cold with focal areas containing small intratumoral TIL infiltrates and/or peritumoral TILs. Tumor-TIL mapping also shows the heterogeneity of tumor immune interactions within in each case, which suggests that TILs might also be clinically useful as a surrogate marker to evaluate tumor heterogeneity.

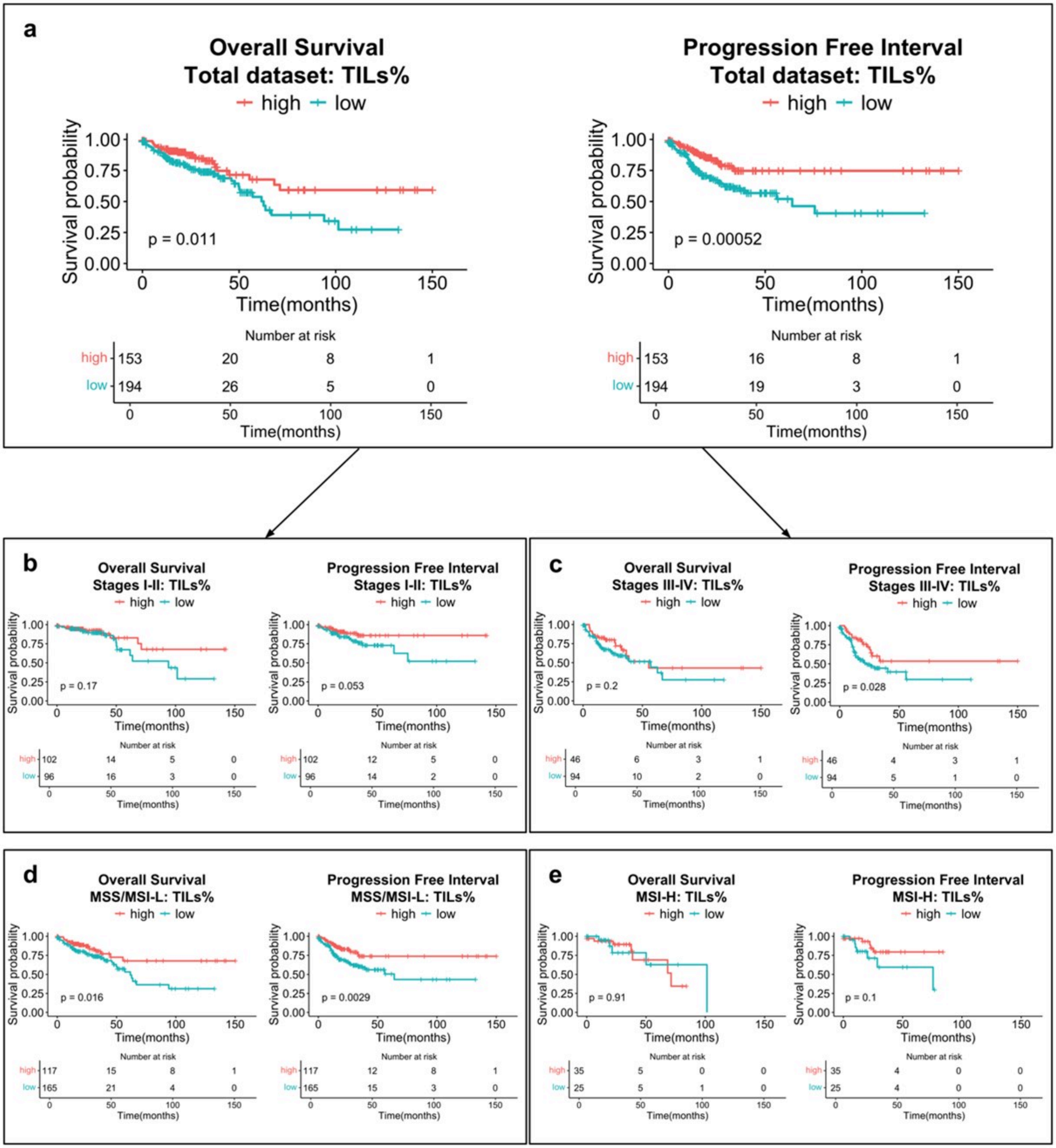

### Figure 4

Stratified Kaplan-Meier analyses with overall survival (OS) and progression free interval (PFI) to evaluate TILs% as a Pathomics biomarker. a) Mean TILs% was used to define high TILs% as >14.1% and Low TILs% as ≤14.1% to study OS and PFI in all TCGA COAD patients. b) High and low TILs% subgroups analyzed in AJCC stages I-II localized disease. c) High and low TILs% subgroups analyzed in AJCC stages III-IV metastatic disease. d) High and low TILs% subgroups analyzed in MSS/MSI-L. e) High and low TILs% subgroups analyzed in MSI-H.

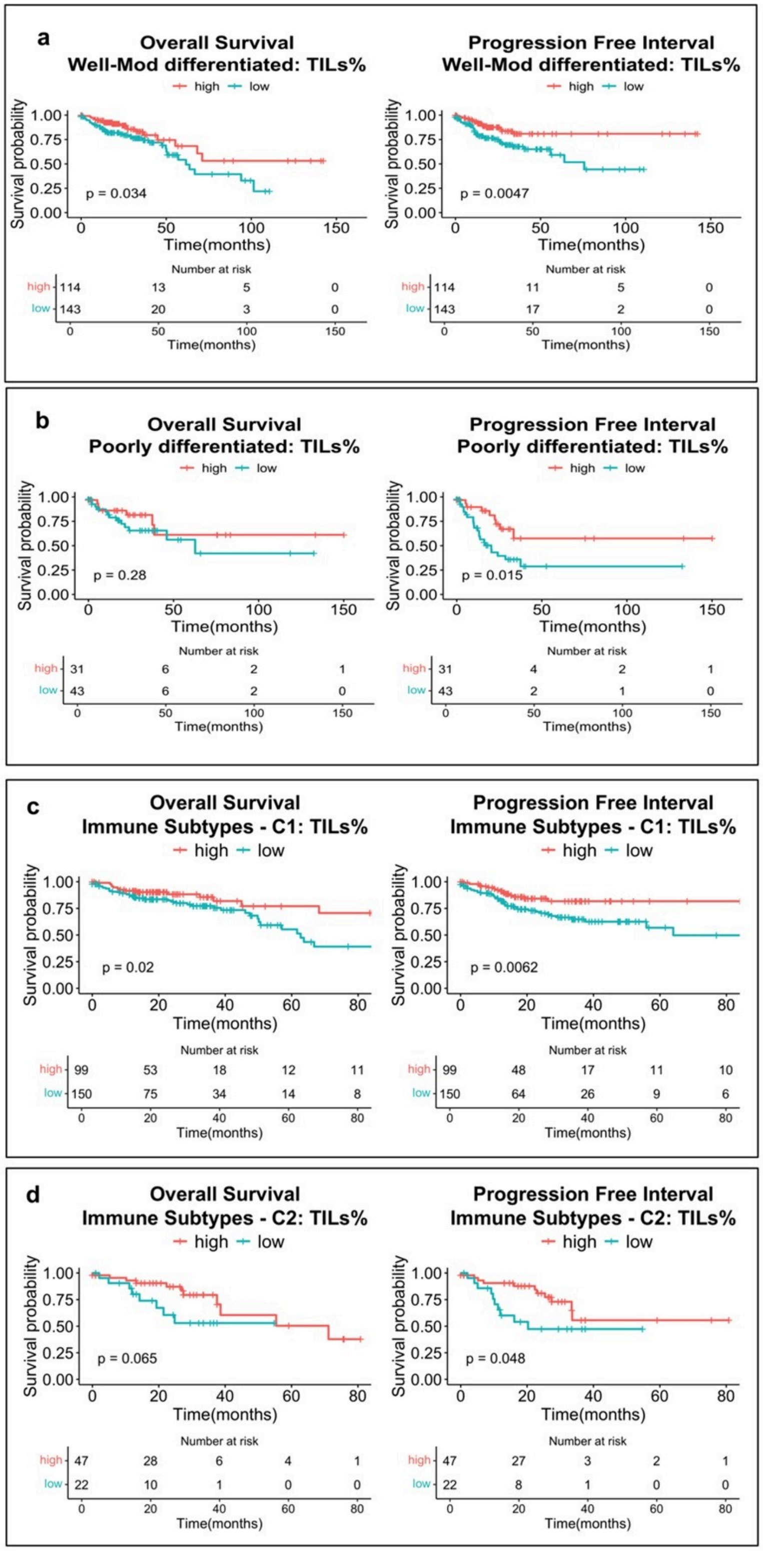

### Figure 5

Kaplan-Meier curves for OS and PFI in histological grades and immune subtypes stratified by TILs% in TCGA COAD. a) Kaplan-Meier curves illustrate OS and PFI for Well to moderate differentiated grade. b) Poorly differentiated grade. c) C1. d) C2. TILs% correlation with C3, C4, and C6 are not shown due to small sample size.

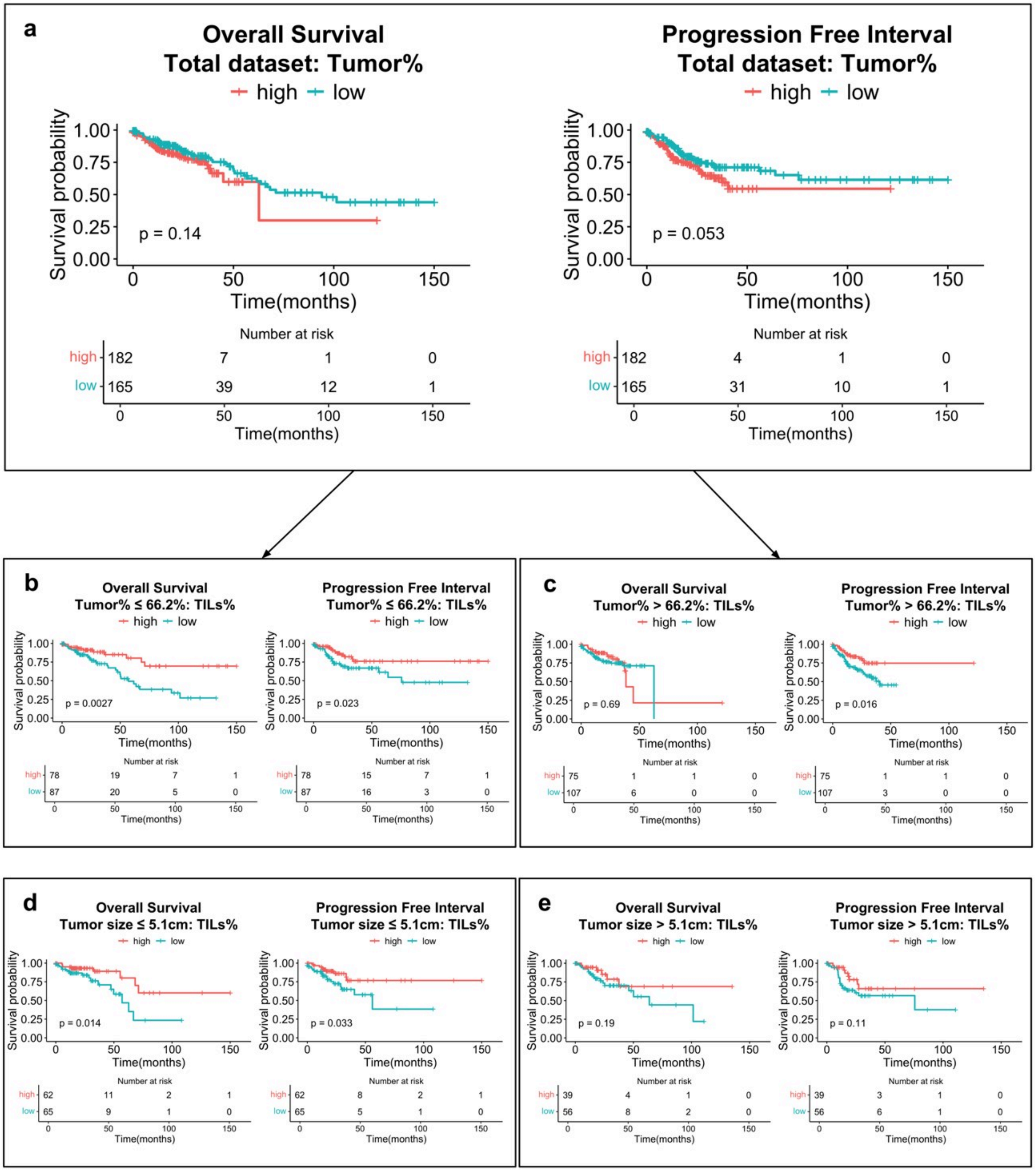

**Figure 6**

Relationship of tumor% and gross tumor size stratified by TILs% on OS and PFI. After stratifying the cohort into high and low tumor% and gross tumor size subgroups, Kaplan-Meier plots show that high TILs% is associated with enhanced OS and increased PFI. a) effect of high vs low tumor% on OS and PFI. b) high vs low TILs% in the low tumor% subgroup. c) high vs low TILs% in the high tumor% subgroup. d) High and low TILs% subgroups in gross tumor size ≤5.1cm. e) High and low TILs% subgroups analyzed in gross tumor size >5.1cm.

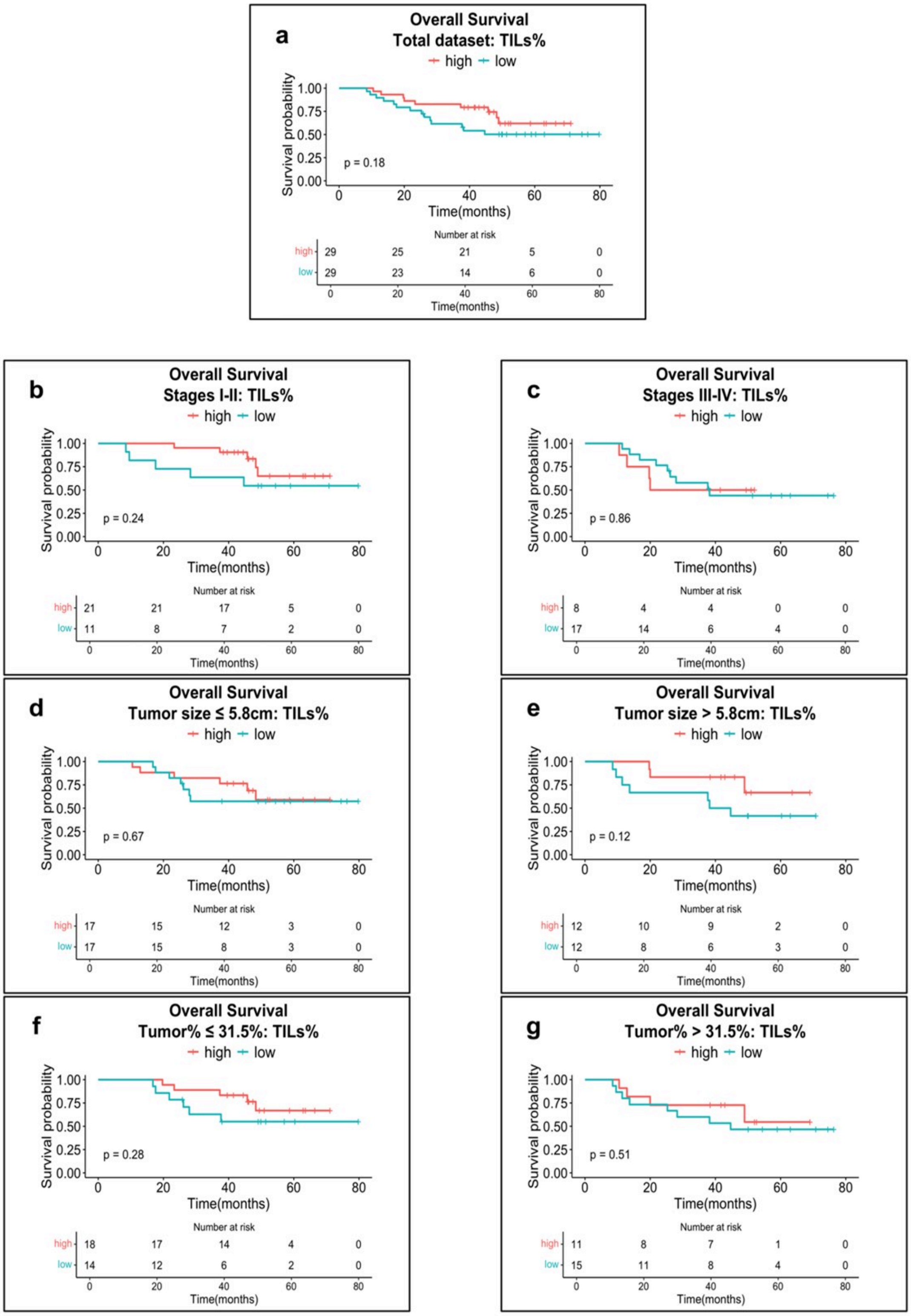

**Figure 7**

Stratified Kaplan-Meier analyses to evaluate TILs% as a prognostic biomarker with OS only, including AJCC stages, gross tumor size, and tumor content in the UKentucky dataset. a) high vs low TILs% in the entire cohort. b) High vs low TILs% in Stages I-II. c) High vs low TILs% in Stages III-IV. d) High vs low TILs% in gross tumor size ≤ 5.8 cm. e) High vs low TILs% in gross tumor size > 5.8 cm. f) High vs low TILs% in low tumor% in WSIs (≤ 31.5%). g) High vs low TILs% in high tumor% in WSIs (> 31.5%). Despite the limited sample size in the UKentucky dataset, we observed similar trends in comparison to the analyses in TCGA COAD.

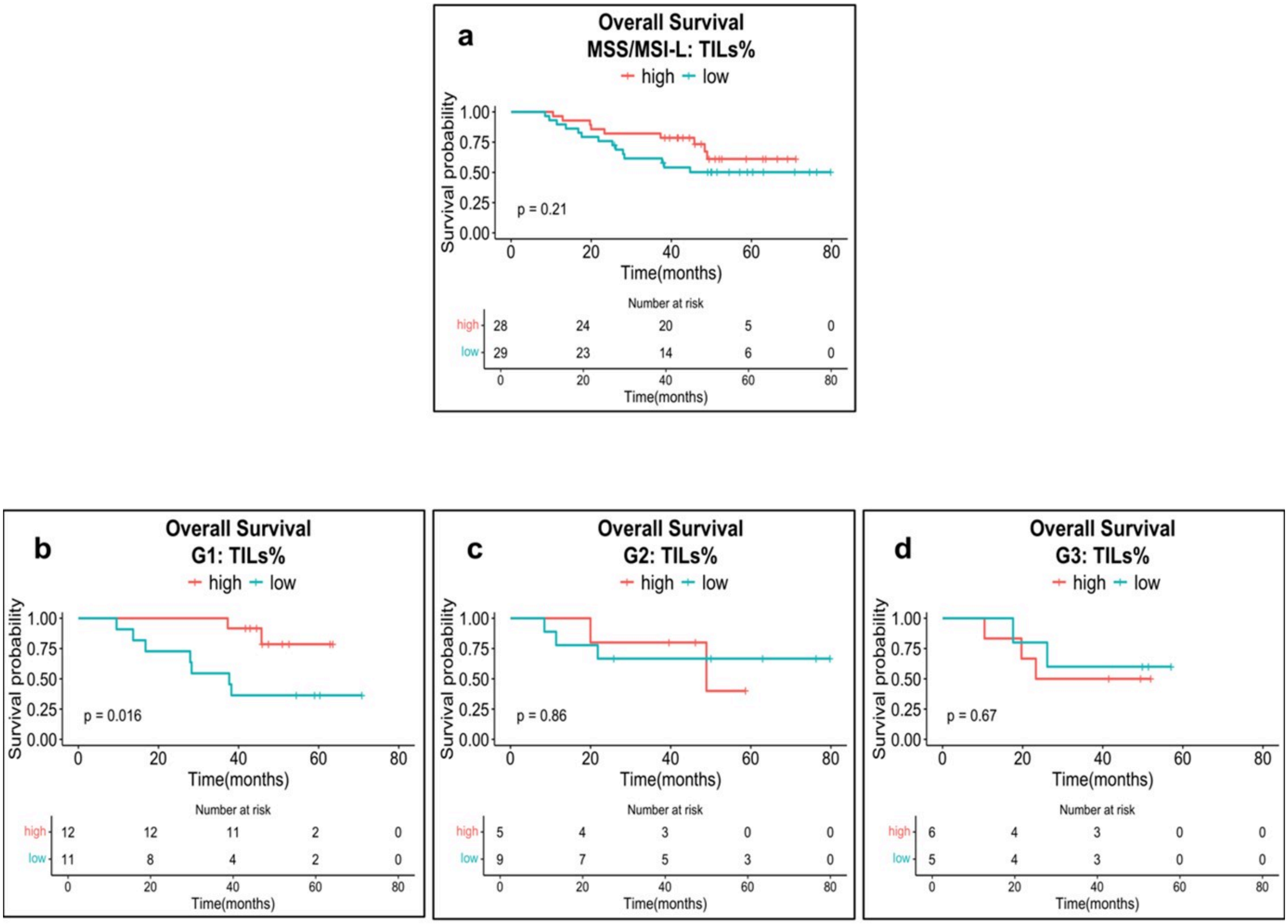

### Figure 8

Stratified Kaplan-Meier analyses to evaluate TILs% as a prognostic biomarker with OS only, including MSI status and tumor budding scores in the UKentucky dataset. a) High vs low TILs% in MSS/MSI -L. b) High vs low TILs% in G1 tumor budding. c) High vs low TILs% in G2 tumor budding. d) High vs low TILs% in G3 tumor budding.

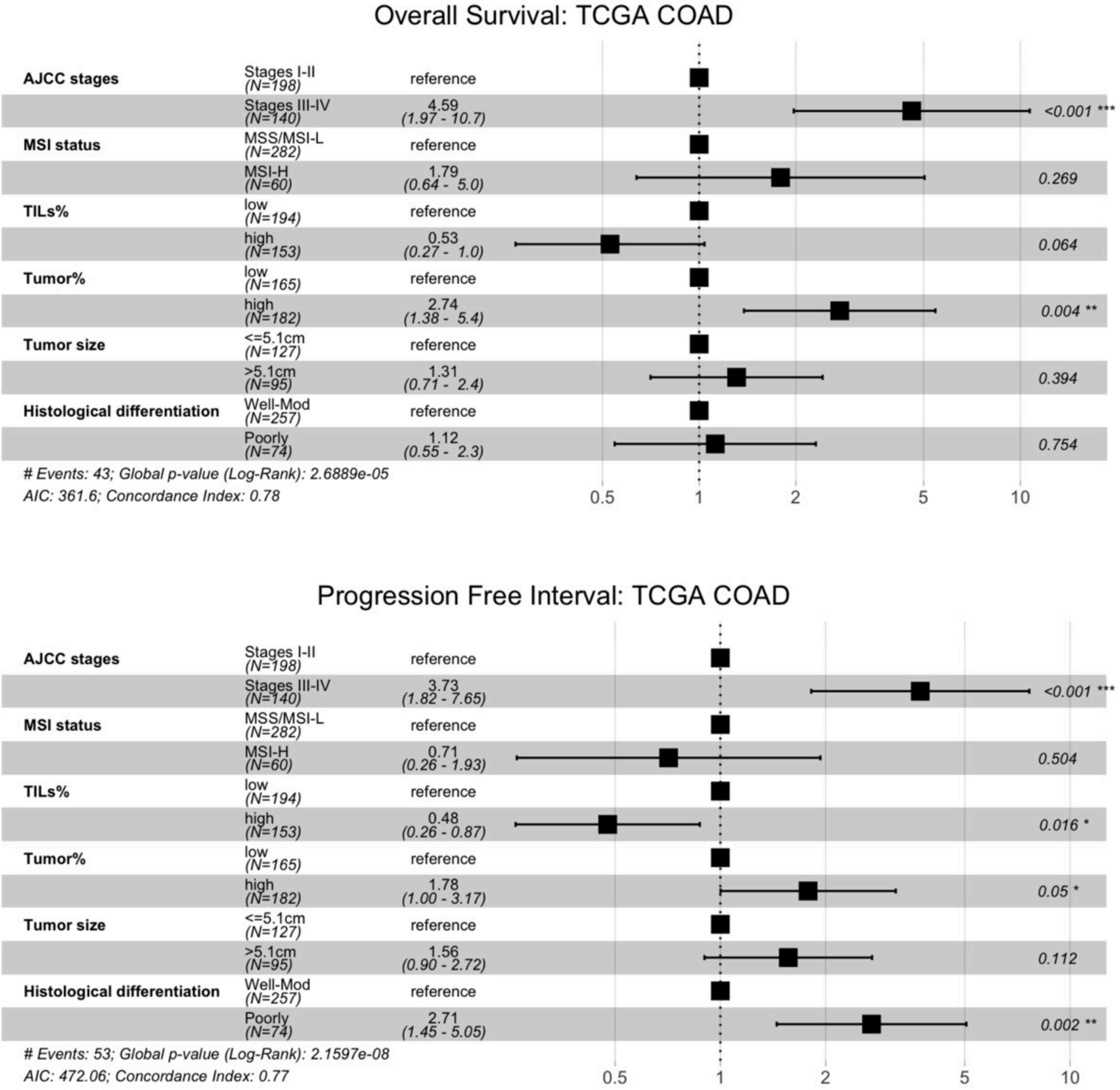

### Figure 9

Multivariate Cox regression analyses in TCGA COAD. Forest plots illustrating hazard ratios for OS (top) and PFI (bottom) in TCGA COAD cohort. AJCC stages III-IV show significantly higher hazard ratios compared to stages I-II for both OS and PFI ($p<0.001$). High TILs% is associated with improved PFI ($p=0.016$). Tumor% is a significant factor for OS ($p=0.004$) and for PFI ($p=0.05$). MSI status and tumor size are not significant predictors for either outcome.

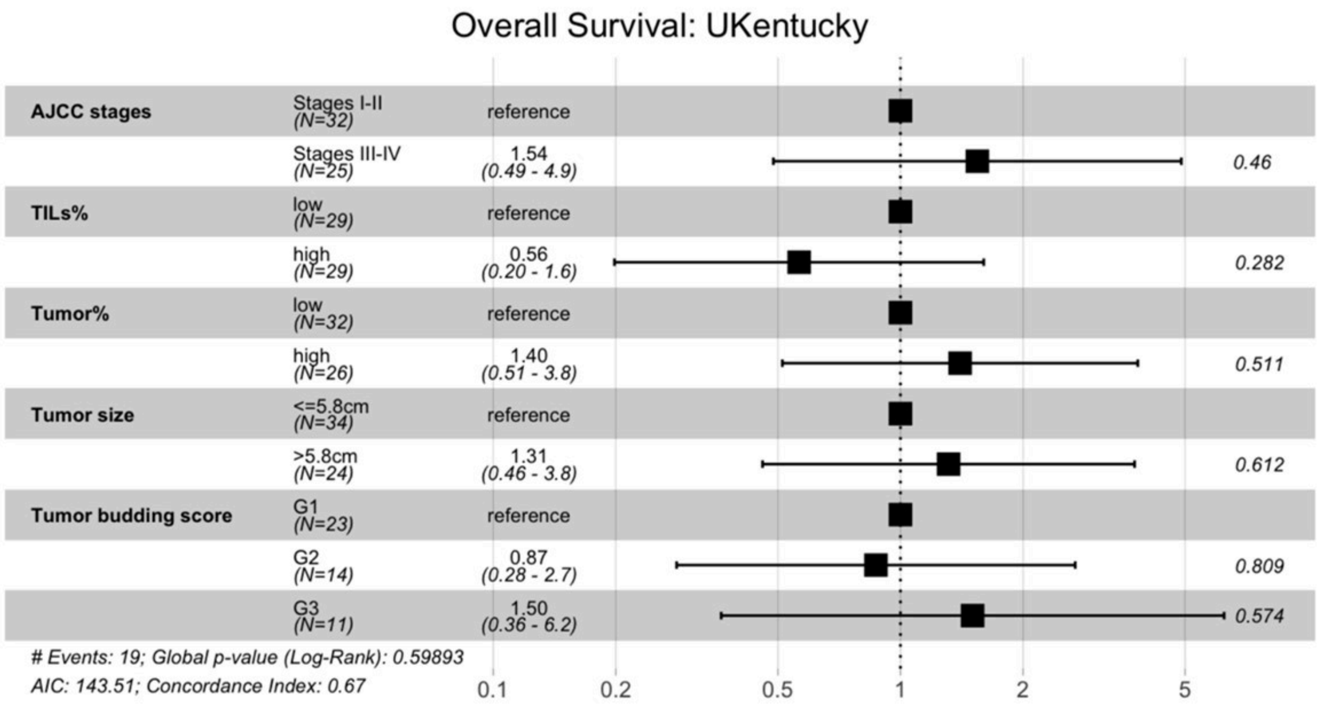

**Figure 10**

Forest plot of hazard ratios from multivariate Cox proportional hazards model of OS in UK dataset. Concordance index: 0.67. # events: 19, global p-value (Log-Rank):0.59893.

## Supplementary Files

This is a list of supplementary files associated with this preprint. Click to download.

- SupplementaryData.docx
